# Ocular accommodation and cognitive demand: An additional indicator besides pupil size and cardiovascular measures?

**DOI:** 10.1186/1477-5751-7-6

**Published:** 2008-08-23

**Authors:** Stephanie Jainta, Joerg Hoormann, Wolfgang Jaschinski

**Affiliations:** 1 Institut fuer Arbeitsphysiologie an der Universitaet Dortmund, Ardeystraße 67, D-44139, Dortmund, Germany

## Abstract

**Background:**

The aim of the present study was to assess accommodation as a possible indicator of changes in the autonomic balance caused by altered cognitive demand. Accounting for accommodative responses from a human factors perspective may be motivated by the interest of designing virtual image displays or by establishing an autonomic indicator that allows for remote measurement at the human eye. Heart period, pulse transit time, and the pupillary response were considered as reference for possible closed-loop accommodative effects. Cognitive demand was varied by presenting monocularly numbers at a viewing distance of 5 D (20 cm) which had to be read, added or multiplied; further, letters were presented in a "n-back" task.

**Results:**

Cardiovascular parameters and pupil size indicated a change in autonomic balance, while error rates and reaction time confirmed the increased cognitive demand during task processing. An observed decrease in accommodation could not be attributed to the cognitive demand itself for two reasons: (1) the cognitive demand induced a shift in gaze direction which, for methodological reasons, accounted for a substantial part of the observed accommodative changes. (2) Remaining effects disappeared when the correctness of task processing was taken into account.

**Conclusion:**

Although the expectation of accommodation as possible autonomic indicator of cognitive demand was not confirmed, the present results are informative for the field of applied psychophysiology noting that it seems not to be worthwhile to include closed-loop accommodation in future studies. From a human factors perspective, expected changes of accommodation due to cognitive demand are of minor importance for design specifications – of, for example, complex visual displays.

## Background

Accommodation of the eye refers to changes in the refraction of the ocular lens in order to provide a sharp retinal image at any viewing distance of the visual target. Accommodation – like the pupil size – is controlled by the autonomous nervous system, predominantly mediated by the parasympathetic branch. However, there is anatomical, pharmacological and physiological evidence for an additional sympathetic input – via adrenoceptors [1,2]. From a human factors perspective, measurements of accommodation can be relevant for two reasons: first, the design of complex visual displays (for example, virtual image displays) may include conditions, where the accommodative response is not appropriate or mislead, i.e. blurred vision may result [3,4]. This question is complicated by the fact that accommodation is affected by factors like contrast, blur or perceived distance [5]. Second, cognitive demand can influence the accommodative response via an activation change in the autonomous nervous system [1,6,7]. One should know how stable and large such "cognitive-induced shifts" in accommodation might be, to evaluate expected blurred vision under high cognitive load. However, the main purpose of the present paper is to evaluate the possibility that shifts in accommodation might be an indicator of the amount of cognitive load imposed on a task operator. In addition to the well-known pupillary response to cognitive demand [8], ocular accommodation as a second ocular indicator may improve the identification and clarification of autonomic activation. This combined approach might be advantageous, because pupillary responses alone have some disadvantages: for example, the pupil is directly dependant on the illumination level, which leads to ceiling effects in dim surroundings; further, the pupil is thought to be an unspecific indicator of autonomic changes – it reflects general states of mood, motivation, emotions and so on. The accommodation, in contrast, might be strictly sensitive to cognitive demand changes and is not directly influenced by surrounding light or involved in homeostatic regulation of the body (which is typically true for other classical autonomic indicators like cardiovascular measures). Modern autorefractors allow for measuring both accommodation and pupil size, even dynamically with frequencies up to 25 Hz; these video techniques measure the eyes from a remote position. The measurement of both indicators – pupil size and accommodation – with a simple video recording system is a tempting possibility, that additionally motivated this paper.

Cognitive demand can affect open-loop accommodation, i.e. when no appropriate stimulus is presented [7,9,10]. Unfortunately, for closed-loop accommodation at near viewing distances – a situation more relevant for human factor applications – the results for cognitive effects are conflicting: Wolffsohn, Gilmartin, Thomas & Mallen (2003), for example, reported no change of accommodation while subjects checked summation-tasks for correctness. Otherwise, Winn, Gilmartin, Mortimer & Edwards (1991) described a mean increase (i.e. a near shift) in accommodation by 0.17 D when subjects had to respond to a target letter rather than reading the letters to themselves. Further, Kruger (1980) showed that the average accommodation increased by 0.28 D when the subjects changed from reading to adding two-digit numbers. On the contrary, Malmstrom, Randle, Bendix & Weber (1980) found a decrease in accommodation when subjects fixated a target and additionally counted backwards, in contrast to pure fixation. Bullimore & Gilmartin (1988) described an accommodative response while numbers were presented in rows and columns: for the 5 D viewing distance, the accommodative responses decreased by 0.04 D with the alteration from reading to adding [11]. The recent study of Davies, Wolffsohn & Gilmartin (2005) used an independent physiological indicator (heart period): a reduction in accommodation with increasing cognitive demand coincided with a reduction in heart period (the correlation of both effects amounted to r = 0.98). Cognitive demand was altered by varying the speed of a two-alternative forced choice task. The change in accommodation and heart period was interpreted as an increase in sympathetic activation in autonomic control of the body. Taken together – no general answer appeared to the question about the effects of cognitive demand on accommodation.

The aim of the present studies was therefore to ensure – for a possible human factor application – that closed-loop accommodation is an indicator of cognitive induced changes in autonomic balance. We tried to clarify the confusing findings of previous research by considering all previously reported information about stimulus conditions [1], instructions [5] or subject's refractive status [12]. Additionally, we considered the effect of two modulating factors: gaze shifts and performance measures, which endanger the correct interpretation of accommodative changes. Cognitive operations could induce different eye movement patterns [13,14]; consequently, possible changes in gaze direction may alter the measured accommodation without any change in curvature of the lens [15-17]. None of the previous research included measurements of gaze direction. Further, in order to confirm that accommodative effects are really induced by cognitive demand, the intended demand has to be validated by means of behavioral changes, i.e. performance measures. Increasing cognitive demand should result in increased performance times or higher error rates. In most of the studies described above, the accommodative response was pooled across correct or false results and no further control of errors was implemented, whereas – usually – the acceptable level of performance should not exceed 25% error rates, when all trials are considered in the calculation of means [18,19]. One should consider that errors might occur due to intermittent blurred vision of the targets: such short-term accommodative far-drifts or stares are somewhat likely in extreme near viewing conditions [20,21]. Thus, in such conditions it remains unclear whether errors are the result of erroneous task processing or not-perceived targets. To avoid such uncertainties, the "cognitive-induced" shift in accommodation should be based on accommodative data collected during correct task performance.

For correct interpretation of possible changes in the autonomic nervous system, we included the following reliable indicators of autonomic balance: heart period or heart rate and pulse transit time as cardiovascular parameters [1,22-27] and the well-documented pupillary response [8,28-33]. We compared these classical indicators of autonomic activation with accommodative effects, looking for evidence of conformity. We collected data during 4 experiments, including prior reported tasks like reading, adding and multiplying numbers and a variation of the "n-back"-task, which is known to demand processes of short-term memory [34,35].

## Results of experiments 1 to 4

### Experiment 1: Reading and adding within a number-matrix

**Figure 1:**
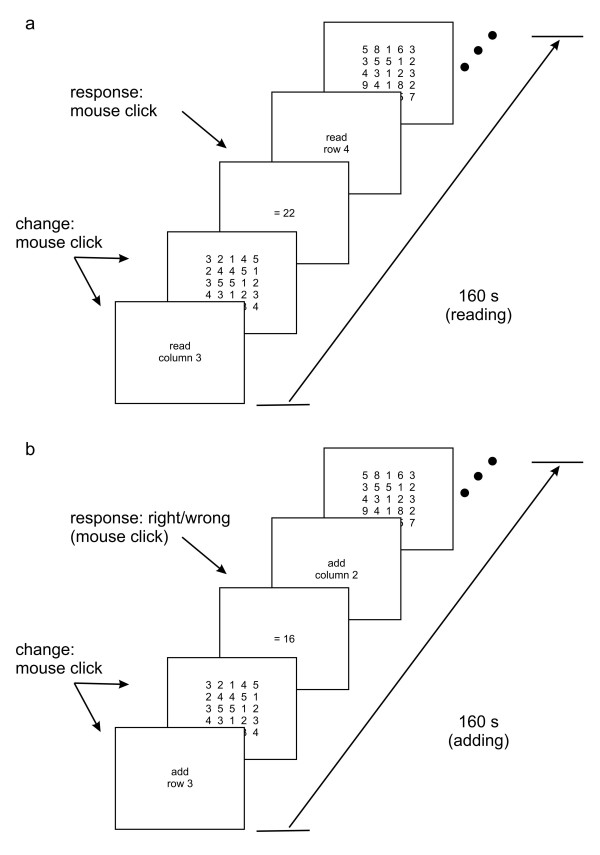
**Time scheme of experiment 1**. In a. the reading and in b. the adding period is shown in time-dependant details.

40 subjects were asked to read or add one-digit numbers arranged in rows and columns of a 5 × 5 number matrix (2.4 deg width × 2.8 deg height; see Figure 1); our task was closely related to the one of Bullimore and Gilmartin (1988). We had periods of reading and adding that lasted 160 s. Reading-adding and adding-reading task sequences were presented and the order was counterbalanced within subjects. After each task sequence of 320 s (160 s reading + 160 s adding, and vice versa) a break of 10 min reduced possible carry-over effects. The instructions "read" or "add" were given at the beginning of each block and indicated which row/column was to add/read. No timing protocol was enforced, so that reading and adding was completely self-paced. For the adding period, a possible result (randomly correct or incorrect by ± 1 in half of the blocks) had to be indicated as correct or not; in half of the subjects, the right (left) button was assigned as "correct" ("incorrect") and vice versa for the other half of the sample. In order to have similar procedures in the adding and reading task, the following response was used after the reading task: randomly, either a "11" or "22" appeared at the end of the task; half the participants had to press the right button for the response "11" and the left button for "22", while the other half had the reversed assignment. Accommodation and gaze direction were measured with the PowerRefractor and the PowerRef II (see General Methods).

### Results of experiment 1

**Figure 2:**
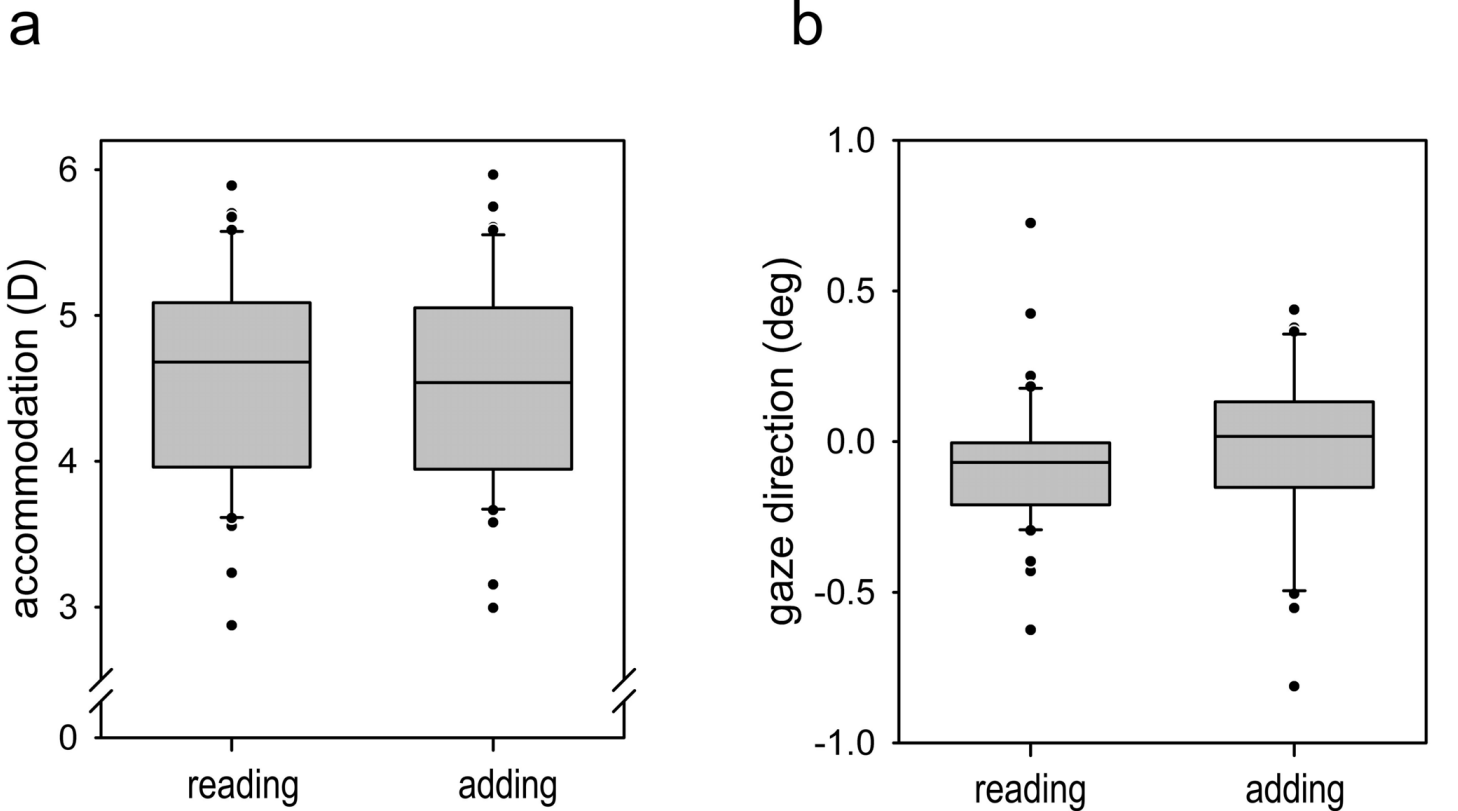
**Box-and-Whisker Plots of the results of experiment 1**. In a. the accommodation response (D) and in b. the gaze direction (deg) as function of task (reading & adding) are shown; both were statistically significant but – as shown in the Appendix – not independent of each other: the measured change in accommodation was mainly induced by the observed change in gaze direction.

The sequence of tasks had no effect, thus the two repetitions were averaged. When changing from reading to adding, the performance data showed a mean decrease in correct responses of 4% (F(1,39) = 7.70; p = 0.01) and the pupil dilated by 0.3 mm (F(1,39) = 36.76; p < 0.01) relative to a diameter of 4.91 mm during reading; these results indicated an increase in task difficulty [36]. Both cardiovascular parameters decreased: the heart period by 13 ms (F(1,39) = 9.09; p < 0.01) and the pulse transit time by 1.27 ms (F(1,39) = 7.31; p = 0.01). According to Weiss, Del Bo, Reichek and Engelman (1980), we calculated a quotient of -1.35 for the change in cardiovascular parameters, indicating an increase in sympathetic activity. Additionally, changing the task from reading to adding induced an apparent decrease in accommodation of 0.07 D (F(1,39) = 4.17; p = 0.04; CI 95%: (-0.15, +0.01)) and a change in gaze direction of 0.14 deg to the right (F(1,39) = 4.42; p = 0.04) (see Figure 2), although the target position was exactly the same for both instructions.

Because of this significant change in gaze direction, we examined the extent to which the accommodative measure depends on gaze direction (see Appendix) and decided, theoretically and data motivated, to consider the gaze direction as covariate for accommodation in the analysis of variance: the resulting accommodative effect by changing the task became non-significant (F(1,38) = 1.52; p = 0.23). We considered the best-fit linear equation between gaze direction and accommodation (see Appendix) to obtain a corrected accommodation level and calculated a 95%-confidence interval (-0.04; +0.01) for the remaining mean change of -0.02 D from reading to adding. Further, only 2% of variance could be explained by individual differences, i.e. by susceptible individuals, in this presumed "cognitive-induced effect" in accommodation, reflected by the interaction "subject × task" [37]. Additionally, the individual accommodative effects were neither correlated with those in pupil size (r = 0.14; p = 0.36), in heart period (r = -0.03; p = 0.84) nor in pulse transit time (r = -0.11; p = 0.49).

In sum, experiment 1 showed no evidence for the "cognitive-induced shift in accommodation". However, the change in correct response was only 4%; thus, our task might have been too easy to provoke an adequate sympathetic reaction in the ciliary muscle. Therefore, in experiment 2 we adopted another, more difficult task from the literature.

### Experiment 2: Reading and adding two-digit numbers

**Figure 3:**
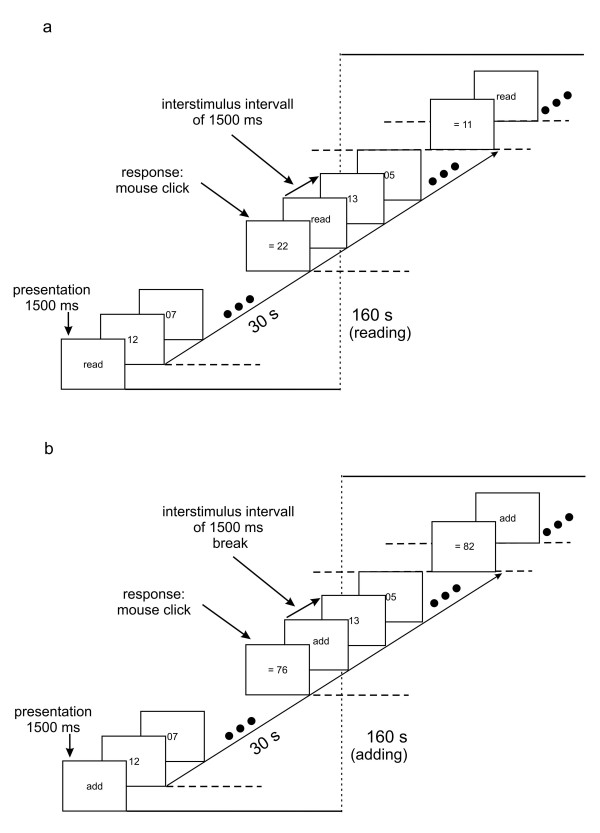
**Time scheme of experiment 2**. In a. the reading and in b. the adding period is shown in time-dependant details.

40 subjects viewed two-digit numbers (see Kruger (1980)); each number was presented for 1.5 s and they were separated by pauses of 1.5 s (see Figure 3). This paced 160 s task period comprised 5 blocks of 30 s and each block contained the presentation of 10 two-digit numbers; these numbers were exactly the same for the reading and adding task and 10 numbers contained six "easy", like 02 or 09, and four "difficult" numbers, like 17 or 14; the latter were restricted to vary between 11 and 19. We had reading-adding and adding-reading task sequences in counterbalanced order. The instructions "read" or "add" were given at the beginning of each 30s-block and after 30 s a possible result was presented. For the reading period the subjects had to quit randomly presented numbers ("11" or "22"), while for the adding period a possible result (incorrect by ± 1 in half of the blocks) had to be indicated as correct or not. Accommodation and gaze direction were measured using the PowerRef II (see General Methods).

### Results of experiment 2

The amount of correct responses decreased by 16% when switching the task from reading to adding (F(1,39) = 50.72; p < 0.01), refecting a higher cognitive demand then in experiment 1. Accordingly [36], the pupil dilated even more, i.e. by 0.5 mm, (F(1,39) = 38.92; p < 0.01; reading pupil size: 4.83 mm). Heart period and pulse transit time decreased by 14 ms and 2.8 ms, respectively (F(1,39) = 6.51; p = 0.01 and F(1,39) = 8.39; p < 0.01) and the quotient of these changes (q = -2.63) indicated an increase in sympathetic activity as in experiment 1 [25]. Generally, the sequence of tasks had no effect, except for a statistically significant interaction of task sequence × task for correct responses (F(1,39) = 9.42; p < 0.01: subjects made 11% more errors when they added first). When the task changed from reading to adding, gaze direction changed by 0.31 deg to the right (F(1,39) = 13.76; p < 0.01) and accommodative raw data decreased by 0.10 D (CI 95%: (-0.19; +0.01)). The latter accommodative effect diminished to 0.03 D (F(1,38) = 3.67; CI 95%: (-0.05; +0.01)), when gaze direction was used as covariate. Further, only about 5% of variance [37] was explained by the variation in "cognitive-induced" effects between the subjects. Again, accommodative effects were neither correlated with changes in pupil size (r = 0.16; p = 0.32), in heart period (r = -0.06; p = 0.71) nor pulse transit time (r = 0.12; p = 0.48).

In sum, the cognitive demand was higher in experiment 2, but, nevertheless, the accommodative change did not reach statistical significance after the change in gaze direction was taken into account.

Unfortunately, in experiments 1 & 2, separate post hoc analyses of correct and incorrect trials – as claimed for in the introduction – were not possible since a task contained a block of number presentations and summarized performance measures. Therefore, we changed the task design in the following experiment 3.

### Experiment 3: Reading, adding and multiplying numbers

**Figure 4:**
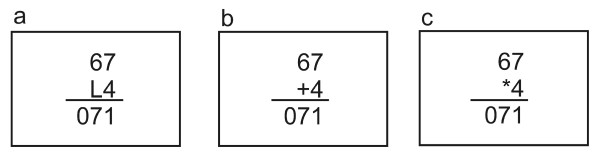
**Task presentations for experiment 3**. The task layout for reading (a), adding (b) and multiplying periods (c), for comparison.

Twenty subjects had to read, add or multiply a two-digit and a one-digit number; number combinations were identical for all three tasks and selected in order to avoid trivial combinations like "20*1". The arrangement of the numbers is shown in Figure 4; for reading the numbers, a "L" or "R" was placed between them and subjects had to react with the left or right mouse button, respectively (see Figure 4). During adding and multiplying periods, the presented result could be incorrect by ± 1 or ± 10; subjects had to quit the result as correct or false. Each number combination was presented for 2 s and the complete reading, adding or multiplying period lasted again 160 s.

We had two reading periods: one before (or after) adding and one before (or after) multiplying periods; task sequences was counterbalanced across subjects. For statistical analysis, we distinguished between an experimental phase – reading versus calculating – and a calculation content – adding versus multiplying.

Accommodation and gaze direction were measured using the PowerRef II (see General Methods). In addition to the averages of 128 s task periods, the accommodation and gaze data were clustered into 8 periods of 20 s to trace accommodative changes throughout the task period.

### Results of experiment 3

The reading and adding task did not produce significant differences in the number of correct responses, cardiovascular parameters, pupil size or accommodation. Maybe the task of adding within 2 s was as easy as reading the numbers. However, the multiplication task induced significantly more errors (by 30%) than the reading task (F(1,19) = 56.84; p < 0.01) and than the adding task (F(1,19) = 53.79; p < 0.01), as shown by simple-effect analysis of variance [38]. The ratio of heart period to pulse transit time showed a relative change from reading/adding to multiplying (quotient: -1.40 and -1.62, respectively) indicating an increase in sympathetic activation [25]; the pupil size increased by 0.7 mm for the same comparison (reading-multiplying: F(1,19) = 19.91; p < 0.01;adding-multiplying: F(1,19) = 14.08; p < 0.01).

**Figure 5:**
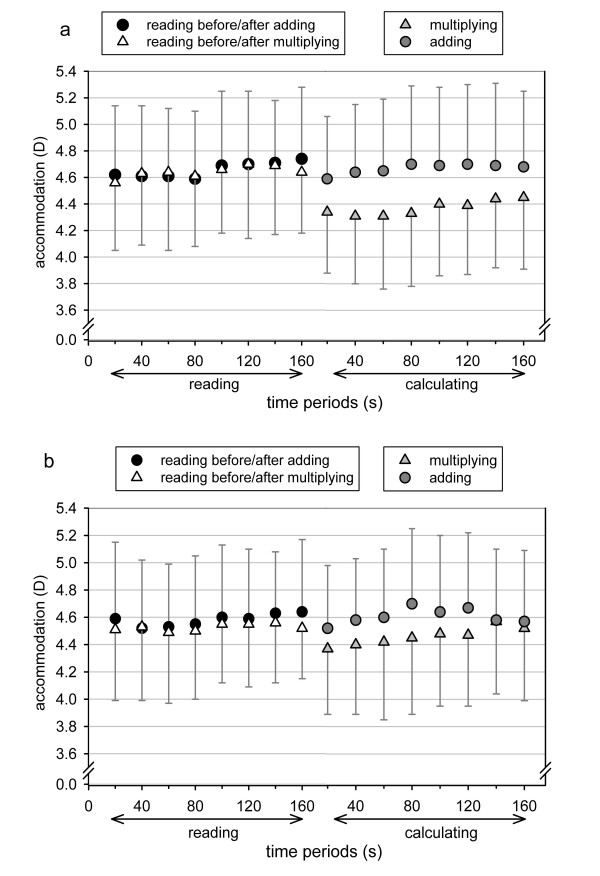
**Accommodation results of experiment 3**. Accommodation (D) as function of experimental phase (reading or calculating) and calculation content (adding or multiplying). a. shows accommodation data for all 20 subjects regardless of the correctness of task processing while b. shows the accommodation data of 16 subjects collected only during correct task processing. (Note that in b the remaining difference in accommodation between multiplying and reading/adding is mostly due to gaze drifts, which were accounted for statistically BUT not graphically.)

In a first analysis, the accommodative data were analyzed considering gaze direction as covariate and irrespective whether responses were correct or incorrect. The analysis of the 8 periods of 20 s showed that accommodation increased slightly by 0.10 D over time during the whole 160 s task period – regardless of the experimental phase or calculation content (F(7,132) = 3.44; p < 0.01). The multiplying task produced a significant decrease of accommodation by about 0.25 D – both relative to the reading task (F(1,18) = 5.94; p = 0.02; CI 95%:(-0.46; -0.08)) and relative to the adding task (F(1,18) = 8.63; p < 0.01; CI 95%: (-0.54; -0.10); see Figure 5a).

For a further analysis, we included only accommodative measures during correct task processing and confined the sample to 16 subjects with an individual mean error rate smaller than 40%. Again, a temporal increase of accommodation during the 160 s periods was observed (F(7,104) = 2.64; p = 0.02). The mean decrease in accommodation during multiplying relative to reading/adding shrank to 0.04 D (CI 95%: (-0.09; +0.01)); the interaction between experimental phase (reading or calculating) and calculation content (adding or multiplying) remained non-significant (F(1,14) = 2.04; p = 0.13; see Figure 5b), indicating no statistical difference between the three tasks for the accommodative response.

In sum, eventhough the cognitive demand varied between reading/adding and multiplying number, experiment 3 showed also no evidence for a "cognitive-induced shift" in accommodation. In the following and last experiment we used a task, which comprised only a single target character – to avoid shifts of gaze direction directly- and induced different levels of cognitive demand.

### Experiment 4: the " n-back" task

**Figure 6:**
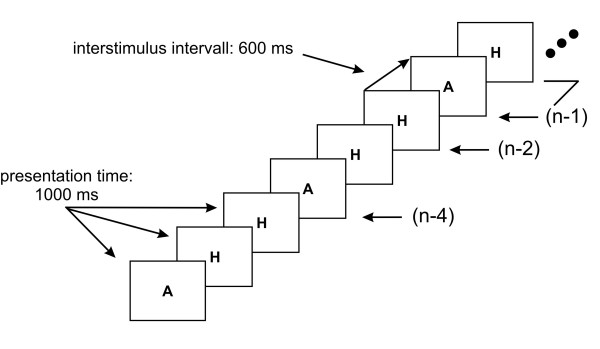
**Time scheme of experiment 4**. The n-back task contained a letter sequence and subjects had to indicate if the letter in the present step "n" was the same as the one before in step "n-1" or "n-2".

Presenting a central target and varying cognitive demand is easily done within an adaptation of the "n-back"-task: a series of characters is presented in random order for 1000 ms (at 600 ms intervals) and the subjects have to indicate whether the letter in the present step n was the same (or not) as the one before in step n-1 (or n-2) [34,35,39] (see Figure 6). By increasing the number of steps backwards, the demand on processes of the short-term memory is increased, indicated by an increase in reaction time and errors. Typically, the reaction time increases mostly by changing the task from n-1 to n-2, whereas the errors continuously increase with n steps backwards [34,35,39]. To our knowledge, the accommodative response to this "n-back" task is not reported elsewhere yet. We started the letter presentation with a short instruction line and three green letters (A or H); then a series of As and Hs was presented for a 160 s period. After each letter a response was given with a button and the reaction time was measured. Mainly, we varied cognitive demand using n-1 and n-2 tasks (N = 20), but had an additional control run with a n-4 task.

To ensure that the measured physiological effects were due to cognitive demand, we compared the lower cognitive demand (baseline: n-1; 160 s) directly with the same or higher demand (task: n-1, n-2 or n-4; 160 s) [40,41], resulting again in 320 s task processing. The task sequences were counterbalanced across subjects. For statistical analysis, we distinguished between an experimental phase (baseline versus task) and a task content (n-1 versus n-2).

We changed the measurement technique for the fourth experiment in order to implement a system which is reported to have an highest standard accuracy of accommodation measurement: accommodation was measured with the open-view autorefractor Shin-Nippon SRW 5000 (Canon Inc., Tokyo, Japan; see General methods). In a separate control experiment, we tested for possible gaze shifts during the n-back task: for 10 subjects we measured the gaze direction with the PowerRef II (described above) while they performed the n-1 and n-2 task. No change in gaze direction between the two tasks was observed (t (9) = 0.56; p = 0.59) and, therefore, for this experiment gaze induced effects on accommodation were unlikely.

### Results of experiment 4

An interaction of experimental phase (baseline vs. task) and task content (n-1 vs. n-2) was significant for errors, reaction time and cardiovascular parameters throughout analyses of variance. We therefore calculated simple-effect analysis of variance [38] to clarify the source of differences: obviously, the n-1-baseline and the n-1-task did not differ significantly. As expected from literature [34,35,39], when the n-2-task was compared with the n-1-baseline the error rate increased by 8% (F(1,19) = 29.63; p < 0.01) and the reaction time increased by 360 ms (F(1,19) = 77.65; p < 0.01).

Additionally, the cardiovascular quotient indicated a decrease in parasympathetic activity between n-1-baseline versus n-2-task (q = -0.24) [25].

For the "n-back" task, average refraction as indicator of accommodative changes varied non-systematically between 3.83 D and 3.91 D (mean SD: 0.28 D) regardless of experimental phases and task contents.

**Figure 7:**
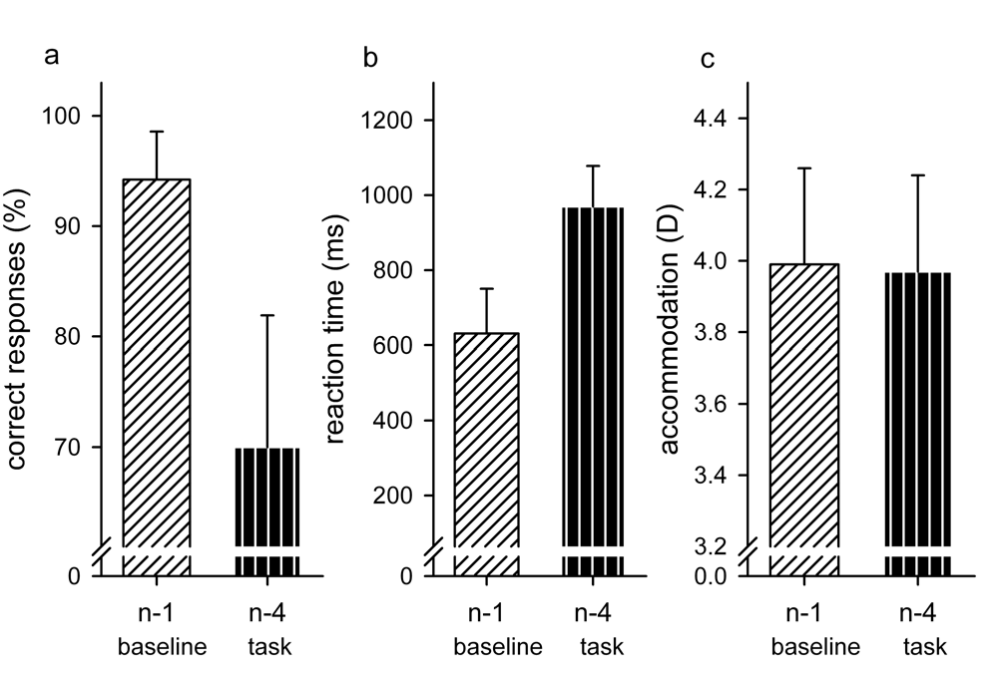
**Results of Experiment 4**. Average results for the additional control block of experiment 4 as function of task content (n-1 or n-4): in a. the amount of correct responses (%), in b. the reaction time (ms) and in c. the accommodation response (D) are shown; SDs are indicated as error bars. Only the amount of correct responses and the reaction time changed significantly with the task content.

In an additional experiment, we applied a larger task demand, i.e. n-4, in a sample of 19 subjects. The results are shown in Figure 7: as expected from previous research [34,35,39], the mean amount of correct responses decreased (monotonously) by 25% (t(18) = 9.79; p < 0.01) when changing the task from n-1 to n-4. Reaction time increased by 340 ms (t(18) = -11.65; p < 0.01) indicating the same increase as for the n-1 to n-2 variation. However, refraction (including accommodation) remained on a constant level of around 3.98 D regardless of the task demand (t(18) = 0.76; p = 0.45).

In sum, in experiment 4 we varied successfully the demand on the short term memory, but accommodation – measured during correct task processing – was still not systematically affected by these demand changes.

## General Discussion

Accommodation depends on parasympathetic and sympathetic innervation of the ciliary muscle [1,12,42,43] and one might expect that accommodation will be influenced by non-optical stimuli, e.g. cognitive demand, just like pupil size or cardiovascular parameters. Since accommodation can recently be measured with commercially available video-based refractors (installed remote from the eyes), we had started this research with the expectation that an easy access to both, accommodation and pupil size, would provide a more complete assessment of the actual activation of the autonomous nervous system – for both, human factor applications and experimental research. Specifically, accommodation was thought to refine the interpretations based on classic autonomic indicators, as pupil size, heart rate, etc.. However, the prerequisite for such an approach is the explanation of previous conflicting results: decreases in accommodation with increasing task difficulty have been interpreted as evidence for a so called "cognitive-induced shift" in accommodation [1,8,12,42,43]. However, increases of accommodation due to cognitive tasks were reported as well [9,44]. In this situation of conflicting literature, we tried to replicate and confirm previous research including classical cardiovascular measures and pupil size [23,36]. As reference, our performance data confirmed task demand variations as well.

In sum, pupil size and performance measures reflected increased task demand throughout all 4 experiments. The same was true for cardiovascular measures – besides the fact, that in experiment 4 a decrease of parasympathetic activity instead of an increase in sympathetic activity with increasing cognitive demand was observed. It remains unclear, why increased demands on short-term memory elicited other autonomic activation pattern than arithmetic tasks.

However, for accommodation, the initially observed decrease was marginally, particularly, after the confounding effect of a cognitive-induced shift in gaze direction was included into analysis [14]: changing cognitive demand resulted in a reliable change in gaze direction which in turn led, for methodological reasons, to systematic errors in accommodation measures (see Appendix). We were able to do an ex-post statistical control of gaze direction which could be measured with our apparatus (PowerRefractor and PowerRef II) for three of our experiments. Moreover, in experiment 3, which contained a more pronounced variation of cognitive demand, a remaining small shift in accommodation after gaze correction disappeared as well, when erroneous trials were excluded from data analysis. In near viewing conditions, it could be that short moments of inattention induce far shifts of accommodation or moments of blurred vision impairs proper target viewing and task performance [20,21]. In any case, it is important that the possibility of unintended influences on the accommodative data (like stares due to, for example, inattention) is reduced when data are collected only during correct task performance. In experiment 4, the performance data showed that the demand on short-term memory was increased as intended by our n-back task variations [34,35,39], but we still did not find a corresponding change in accommodation – no "cognitive-induced shift" occurred.

For all data sets, the partly observed minor (non-significant) accommodative changes were neither correlated with cardiovascular changes nor pupil size variations; this observation confirms the disbelief that our subtle accommodative shifts were mediated by autonomic functions.

Last but no least, reducing our initial sample size of 40 subjects in experiment 1 & 2 to remaining 20 subjects in experiment 3 & 4 (19 subjects for the control run), may have questioned the statistical power. Therefore, we calculated post-hoc power estimates for all our analysis of variances [45]; we found all power values to be larger than 0.95 with one exception of 0.60 for the t-test in experiment 4, where we compared the results of accommodation for n-1 task with the n-4 task. Nevertheless, we conclude, that sample size was adequate to reveal accommodative changes, if they were of physiologically relevant size and inherent in our data.

## Conclusion

Our data showed that the variation of gaze direction and the correctness of task responses might have contributed – probably – to the inhomogeneity of previous results besides other aspects as instructions [5] or initial pupil sizes (corresponding to different depth-of-focus conditions). Finally, our data leave us to doubt changes in closed-loop accommodation due to cognitive demand – at least in near viewing conditions with visually presented targets; at longer viewing distances, effects are even less likely. For practical application, we can draw the positive conclusion that operators using visual displays are unlikely to experience blurred vision due to cognitive demand since we did not find evidence for changes in accommodation that reached practically relevant levels. Although the expectation of accommodation as possible autonomic indicator of cognitive demand was not confirmed, the present results are informative for the field of applied psychophysiology noting that it seems not to be worthwhile to include closed-loop accommodation in future studies.

## Methods

### Targets and subjects

The targets were composed of numbers or letters and were presented on a TFT screen as black on white numbers with a mean background luminance of 30 cd/m^2^. Each number/letter subtended 0.29 deg × 0.37 deg (width × height) at a viewing distance of 5 D (20 cm; as suggested by Gilmartin (1988)). We presented the targets at this very close viewing distances (accommodation demand relative to the individual resting state > 4 D) in order to elicit a strong initial parasympathetic response and to ensure accommodative changes per se. The surrounding room lighting was adjusted individually in order to set the initial pupil size at an individual intermediate size; resulting room lighting varied between 2 and 15 lux.

In general, we used tasks most prevalent in the literature: reading, adding or multiplying of numbers and a "n-back" task. Each task period lasted 160 s; when different tasks were compared with each other, these task periods were presented without any time gap to avoid artefacts due to normal physiological variations of autonomic parameters during homeostasis [41,46]. Additionally, to avoid short time effects of orientation or attentional shifts at the moments of task switching, the first 32 s of each task period were excluded from data analysis and all parameters are described as means across the remaining 128 s. Analyses of variance with repeated measures (with Greenhouse-Geisser adjusted error probabilities) were then calculated.

For each task period, the percentage of correct responses was taken as general performance measure; subjects were told to exert equally effort on focusing the numbers/letters and performing as correctly as possible.

Participants were selected from a pool of 40 male (mean age ± SD: 23 ± 3.6 years), almost emmetropic subjects (best spheres and astigmatic component did not exceed 0.5 D) – with a visual acuity > 0.8 (in decimal units) in the right eye. The near point of accommodation was typical for this age, i.e. close to 10 D on average, and the mean (± SD) of resting accommodation (dark focus) was 0.79 D (± 0.27). Each subject gave informed consent before experiments; the research followed the tenets of the Declaration of Helsinki.

### Measurement of accommodation, gaze direction and pupil size

Dynamic photorefractor measurements: In experiments 1, 2, and 3, accommodation, pupil size and gaze direction were measured objectively and dynamically (25 Hz) using a remote, automatic eccentric infrared photorefractor, the PowerRefractor (MultiChannel) in its standard version [47-49] and its successor, the PowerRef II (PlusoptiX) [50]. No difference between readings of the two photorefractors for different subject subgroups was found, so that the data were pooled. In the current study, all measurements of accommodation reflect sphere data determined in the vertical meridian of the right eye under conditions of monocular viewing. The horizontal gaze direction was determined from the position of the corneal Purkinje image of the refractor with respect to the pupil centre. The PowerRefractor and the PowerRef II are specified to have an accuracy of 0.25 D (for pupil size within 3 – 11 mm) [51]. In order to keep the required fixed 1 m distance from the eyes to the video camera and to present the targets at 5 D, we placed the camera above the subject's eye level and used two dichroic mirrors, which pass visible light and reflect infrared light [52]. The camera was placed in line with the right eye and a chin and forehead rest was used.

During data screening, blink artefacts were removed from the accommodation, pupil size and gaze direction records by eliminating all data points within an interval of 100 ms before and after each eye blink.

Autorefractor measurements: In experiment 4, accommodation was measured with the open-view autorefractor Shin-Nippon SRW 5000 (Canon Inc., Tokyo, Japan) in its standard version [17]. On pressing a button at the joystick of the SRW 5000, the instrument can take static measurements of refractive error in the range of ± 22 D in steps of 0.125 D. When the button is kept pressed, about 45 static readings can be collected within 1 min (data analysis is performed in 0.15 s). A built-in display provides an image of the pupil to allow alignment of the instrument with respect to the subject's visual axis; refractive data were transmitted to a PC [53]. The manufacturer's designation of a minimal pupil size is 2.9 mm. Accommodative data were corrected for outliers (beyond a ± 4 SD range).

### Measurement of electrocardiogram (ECG) and pulse transit time (PTT)

All cardiovascular data passed an eight pole Bessel-lowpass-filter with a corner frequency of 220 Hz. The sampling rate for each channel was set to 500 Hz. To determine the heart period (HP) we used a bipolar record (Einthoven II). The ECG-signal was amplified (Siemens Mingograf 710) and filtered with a time constant of 3.2 s. To identify the inter-beat-intervals (IBI) out of the ECG signal, the QRS complex was determined by a template-matching-algorithm which consisted of a QRS complex template: a mean of 3 to 4 selected intervals of 27 ms after and before the R-wave was calculated and a cross-correlation function identified the remaining QRS complexes. Artefacts were selected automatically [23] and visually by checking the distribution of the IBI for outliers. The resulting mean IBI represented the mean heart period. The pulse transit time (PTT) was defined as time between the R-wave of the QRS-complex of the ECG signal and the initial upstroke of the pulse curve at a peripheral site. The arrival of the pulse in the left pointing finger was measured with a special photoplethysmograph. The signal of the PTT was low-pass filtered with 24 Hz. The upstroke of the pulse curve was detected by an algorithm selecting the minimum of the peripheral pulse curve in an interval of 300 ms after the appearance of the R-wave, which was already determined by the QRS-algorithm. Outliers were defined as values being greater than 260 ms or smaller than 140 ms.

According to Weiss, Del Bo, Reichek and Engelman (1980), we focused on calculating a quotient for the change in cardiovascular parameters, indicating selective branch activity due to a shift in autonomic balance. This quotient (ΔPulseTransitTime/ΔHeartRate; we transferred heart period in heart rate) reflects relative changes of both parameters and is based on a general increase in heart rate accompanied by a decrease in pulse transit time; when this quotient is more negative than -0.9, increased beta-sympathetic activity is deduced, while for a quotient more positive than -0.9 a decreased parasympathetic activity is supposed.

## Competing interests

The authors declare that there is only one competing, academic interest: we are aware of the fact that we replicated accommodative changes, which other researchers published as "cognitive-induced". However, these effects disappeared, after possible alternative effect sources (gaze changes and the correctness of task processing) were taken into account. Trying for publication of these results on the edge of two research disciplines, i.e. human factor research and optometry/vision research, unfortunately, sometimes led to comments as "not of interest for our community" with this particular negative result. A more general journal (on negative results) may be appropriate so that other researchers can take advantage of our experience.

## Authors' contributions

SJ conceived the design, made the final statistical analysis of the data and drafted the manuscript. JH carried out the data aquision and statistical analysis of the cardiovascular data. WJ participated in the design of the study and has been involved in drafting the manuscript. All authors read and approved the final manuscript.

## Appendix: Measures of accommodation at different horizontal gaze directions

**Figure 8:**
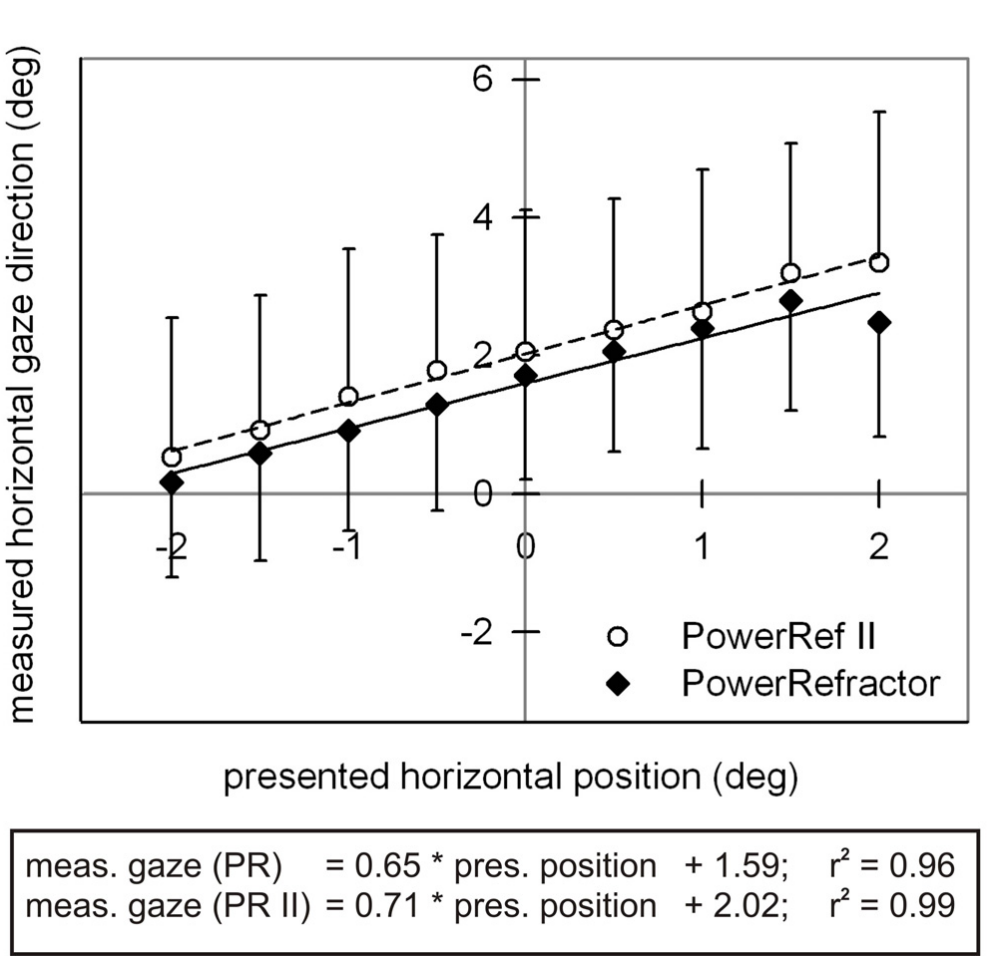
**Gaze direction measurements**. Measured horizontal gaze direction (deg; mean ± SD) as a function of presented horizontal position (deg) for the PowerRefractor and PowerRef II. Regression equations are shown separately. As indicated by the slope of the regression equations, the distance between the presented horizontal positions was underestimated by the measured gaze direction, even though the measurement distance of 1 m was correctly maintained; maybe the Hirschberg ratio set by the default routine was to low for our sample.

The direction of gaze may affect the measured refraction value without any change in curvature of the lens [15-17]. In order to quantify this possible measurement artefact, we presented small targets (0.16 deg × 0.16 deg) at 9 horizontal gaze positions to the right eye at eye level. The total range of presentation was ± 2 deg with gaps between each gaze position of 0.5 deg. Refraction and gaze direction were measured monocularly for 10 subjects with the PowerRefractor and for 15 subjects with the PowerRef II (subject pool described above). Each gaze position was fixated for 2 s and refraction and gaze direction were sampled with 25 Hz; for analysis we extracted the medial second of each stable fixation by cutting off the first and last 500 ms. The measured gaze positions reflected the presented gaze positions (see Figure 8); the deviation of both regression lines along the y-axes reflected a mean difference in the position of the two cameras (PowerRefractor/PowerRef II); both cameras were fixed in 1 m distance – relative to the right eye. Additionally, the stimulus range of ± 2 deg was not completely reflected by the measured data.

**Figure 9:**
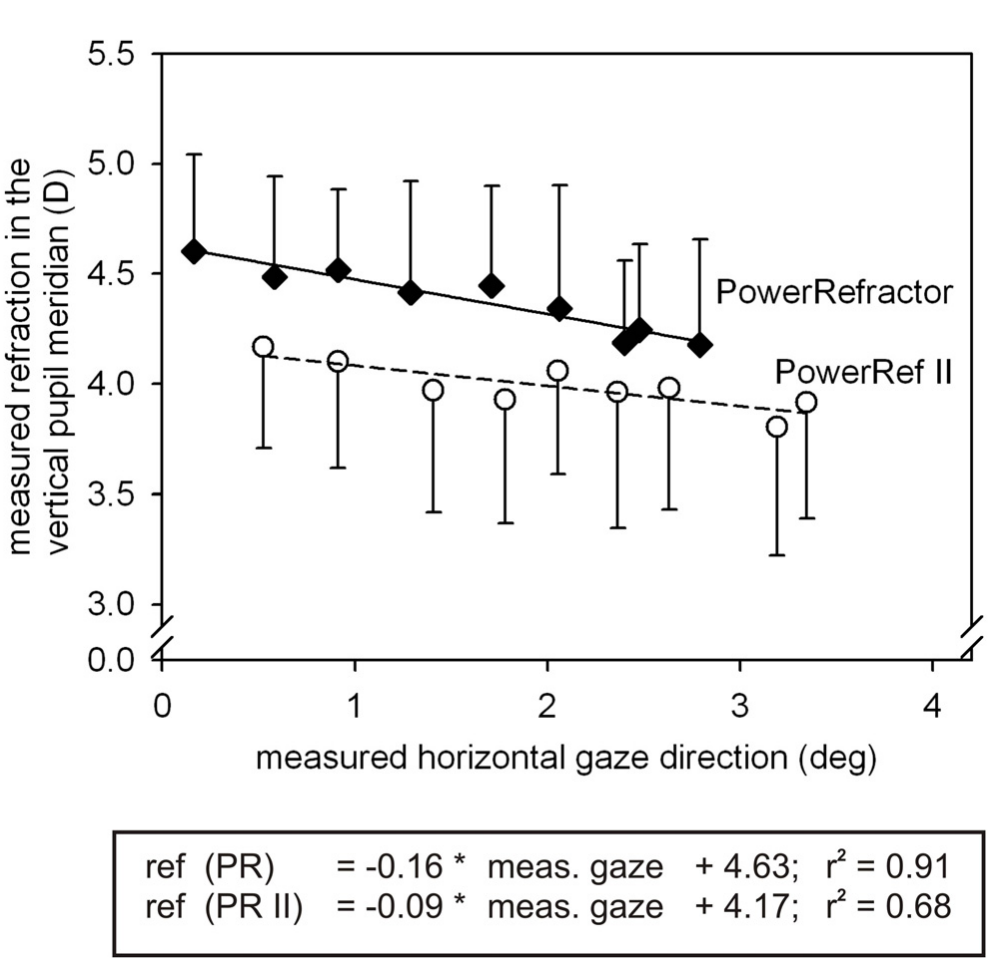
**Accommodation measures (PowerRefractor/PowerRef II)**. Measured refraction in the vertical pupil meridian (D; mean ± SD) as a function of measured horizontal gaze direction (deg) for the PowerRefractor and PowerRef II. Regression equations are shown separately.

More important, the measured refraction decreased by 0.16 D (PowerRefractor) and by 0.09 D (PowerRef II) when the measured gaze direction changed by 1 deg to the right (see Figure 9). This indicated an artefact: off axis refractive errors where directly influenced by gaze direction; therefore, gaze direction should be considered in the statistical analysis of refraction data (as indicator of accommodation).

**Figure 10:**
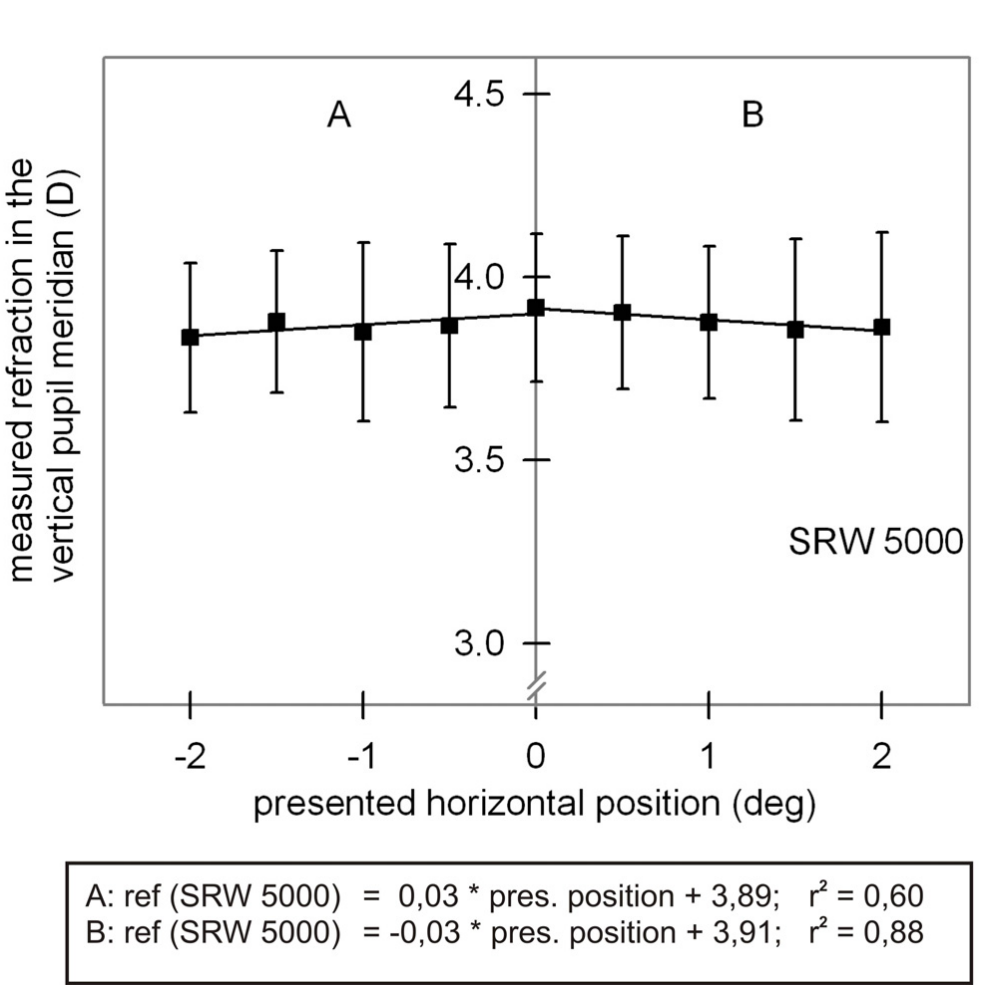
**Accommodation measures (SRW 5000)**. Measured refraction in the vertical pupil meridian (D; mean ± SD) as a function of presented horizontal position (deg) for the SRW 5000. Regression equations are shown separately.

Most studies on "cognitive-induced shifts" in accommodation (reported in the Introduction) used a different technique for measuring accommodation, an autorefractor like the Shin-Nippon SRW 5000 (Canon Inc., Tokyo, Japan; [17]); therefore, we investigated whether the standard version (6.15) of this device also produces the gaze direction artefact. In the same experimental setting, 10 subjects fixated each gaze position until 20 readings of the SRW 5000 were collected. For calculating an average refractive value, the first five and last five readings were excluded. As shown in Figure 10, for gaze changes from central to the right and to the left, the refraction measures decreased with a slope of 0.03 D/deg.

Even though these changes are very small, one should bear in mind that the SRW 5000 provides no opportunity to control for gaze changes. Minor changes in refraction as indicator of possible accommodative changes may reflect gaze changes even when the optic of the refractor is permanently adjusted to reach alignment of the instrument with respect to the subject's visual axis, as it was in our experiments.
